# Leber's idiopathic stellate neuroretinitis: A clinical case

**DOI:** 10.1016/j.amsu.2022.103491

**Published:** 2022-03-21

**Authors:** O. Nabih, L. Arab, L. EL Maaloum, B. Allali, A. EL kettani

**Affiliations:** a Medical Resident at Pediatric Ophthalmology Department, Ho^pital 20, Aou^t, 1953, Casablanca, Morocco; b Professor- Pediatric Ophthalmology Department, Ho^pital 20, Aou^t, 1953, Casablanca, Morocco; c Professor and Head of Pediatric Ophthalmology Department, Ho^pital 20, Aou^t, 1953, Casablanca, Morocco

**Keywords:** Neuroretinitis, Optic nerve oedema, Macular star, Leber's neuroretinis

## Abstract

**Introduction:**

In 1916, Leber's idiopathic stellate neuroretinitis (LISN) was described by Theodore Leber as a rare disease characterized by optic disc swelling associated with a macular star. This fundus appearance can have multiple causes but the etiology of Leber's idiopathic stellate neuroretinitis remains unknown.

**Case report:**

A 40 year-old man consulted for a progressive decline in visual acuity and a blurred vision in his left eye. Corrected Visual acuity of the left eye was hand motion, Funduscopy of the left eye revealed a stellate maculopathy with loss of foveolar depression and a normal optic disc. The angiography confirmed an optic disc oedema. Laboratory investigations were normal. No infectious nor inflammatory etiology was found. Brain imaging was normal. Patient received 3 days of intravenous methylprednisolone **at 10mg/kg/D for 3 days in a row and an oral relay was started with a progressive degression over 2 weeks.** The evolution after treatment was satisfactory, the visual acuity 3 weeks after the intravenous injection of corticoids increased to 2/10.

**Discussion:**

Leber's idiopathic stellate neuroretinitis (LISN) is a disorder characterized by disc oedema, peripapillary and macular hard exudates and, often, the presence of vitreous cells. The changes in the optic nerve are the primary cause of reduced vision in this condition. The more common treatable causes must be excluded wich are cat scratch disease (CSD) and vascular disease. 50% of cases have no identifiable cause and are labeled idiopathic neuroretinitis. There is no consensus regarding optimal treatment. The prognosis of Leber's idiopathic stellate neuroretinitis is good in most cases.

**Conclusion:**

The cause of neuroretinitis must be aggressively pursued before a diagnosis of lebre's idiopathic neuroretinis can be retained in order to formulate an appropriate treatment strategy.

## Highlights

• Leber's idiopathic stellate neuroretinitis is a rare disease characterized by optic disc swelling associated with a macular star.
• The more common treatable causes must be excluded wich are cat scratch disease (CSD) and vascular disease.
• There is no consensus regarding optimal treatment.
• The prognosis of Leber's idiopathic stellate neuroretinitis is good in most cases.

## Author contribution

O.Nabih: drafting the article, study concept, writing the article. L.Aarab: acquisition of data. L.El maaloum: study design. B.Allali: revising the article. A. El kettani: final approval.

## 1 Introduction

In 1916, Leber described an idiopathic stellate retinopathy that he distinguished from the retinopathy of hypertension [1]. The salient characteristics of this condition, as initially described by Leber, are: 1-unilaterality; 2-stellate macular exudate; 3-spontaneous resolution; and 4-unknown etiology [2]. It occurs classically in the 30–40 year age range but also as frequently in children [3]. It is usually idiopathic, but infectious causes, such as syphilis, Lyme disease, cat-scratch disease, toxoplasmosis and viral infection, should be considered [4]. Cases of retinopathy with star formation secondary to known causes are numerous, but few cases of idiopathic Leber's stellate retinopathy are described in the literature. Therefore, we report the case of a patient in whom the diagnosis of disease has been retained.

This study has been reported in accordance with the SCARE criteria [5].

## 2 Case report

**Fig. 1:**
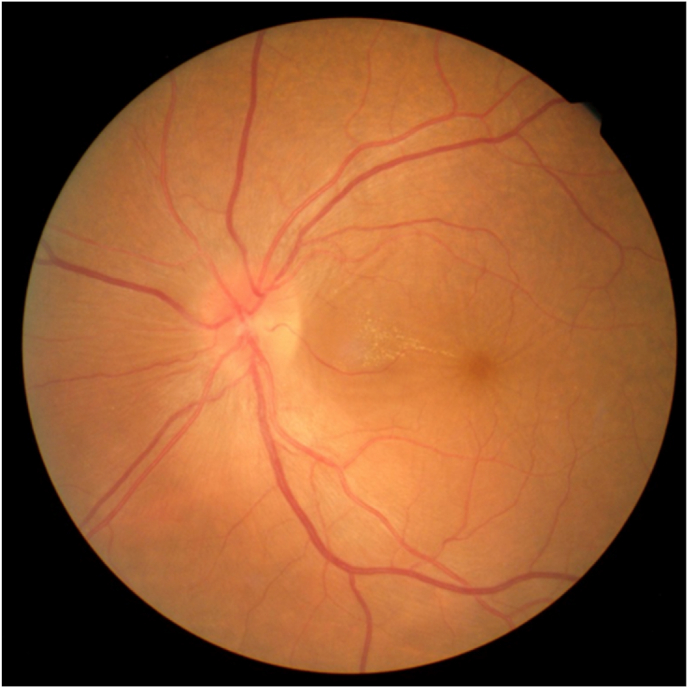
Funduscopy of the left eye revealed a stellate maculopathy with loss of foveolar depression and a normal optic disc.

A 35-year-old man, consulted for a declining of visual acuity. He had a 2-months history of blurred vision in his left eye without pain or redness. The man's corrected visual acuity was 3/10 in the right eye and hand motion in the left eye. The intraocular pressure was 13 mmHg in both eyes. Slit-lamp examination showed no anterior segment abnormality beside a bilateral incipient cortical cataract. Funduscopy of the right eye showed an epiretinal membrane that slightly pulls on the retina associated with macular folds, and a normal optic disc. Funduscopy of the left eye revealed a stellate maculopathy with loss of foveolar depression and a normal optic disc. Figure [1].

**Fig. 2:**
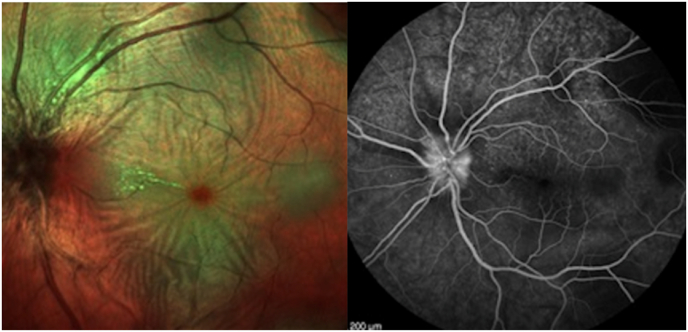
Fluorescein angiography showed an optic disc swelling and no abnormalities nor in the choroidal filling nor in the retinal vessels.

The earlyphase of fluorescein angiography showed no abnormalities nor in the choroidal filling nor in the retinal vessels. An optic disc swelling was confirmed in the left eye by observing fluorescein diffusion from the disc, especially along the vascular axes and indistinct borders in the late phase. Figure [2].

**Fig. 3:**
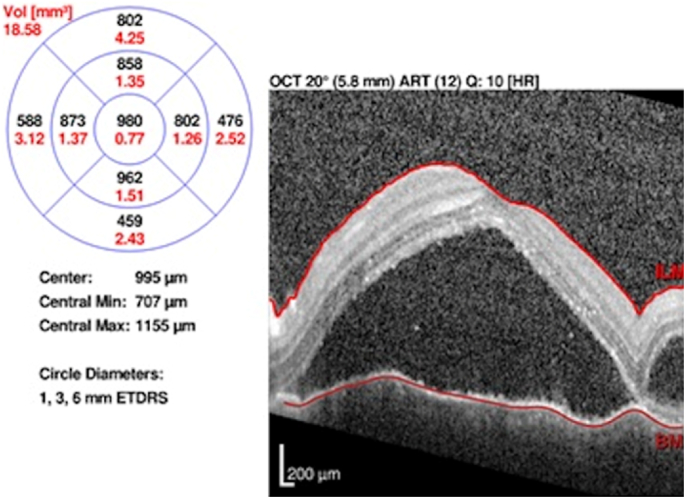
Optical coherence tomography showed a retinal serous detachment with thickening of the retinal pigment epithelium and a few exudates of the IS/OS junction layer.

Optical coherence tomography showed in the right eye an epimacular membrane with thickening of the retinal pigment epithelium and in the left eye, a retinal serous detachment with thickening of the retinal pigment epithelium and a few exudates of the IS/OS junction layer. Figure [3].

The general clinical examination was unremarkable. The results of laboratory investigations did not reveal any inflammatory syndrome, blood count electrolytes, C-reactive protein, and erythrocyte sedimentation rate, were normal. Serologies for cat scratch disease (ELISA for Bartonella hanslae), syphilis (VDRL and fluorescent treponemal antibody-absorption tests), and toxoplasmosis (serologic testing for anti Toxoplasma IgG and IgM), were negative. Tests for tuberculosis including chest radiography gave negative results too. A magnetic resonance imaging (MRI) of the brain was requested but no intracranial lesions were found. The diagnosis of idiopathic Leber's stellate neuroretinitis was retained. Therapy with methylprednisolone at 10mg/kg/D for 3 days in a row and an oral relay was started with a progressive degression over 2 weeks. The visual acuity, 3 weeks after the corticosteroid treatment, improved to 2/10. The prognosis in this case was good. Indeed, after 1 year, the visual acuity remained the same with a clear regression of the stellar maculopathy and the optic disc oedema.

## 3 Discussion

Neuroretinitis (NR) is defined as inflammation of the anterior optic nerve and peripapillary retina [6] The inflammatory process involves exuberant leakiness of the optic disc vasculature, which produces peripapillary serous detachment followed about 10 days later by formation of hard exudates that track towards the macula in a characteristic star pattern [7]. Leber's idiopathic stellate neuroretinitis (LISN) is a disorder characterized by disc oedema, peripapillary and macular hard exudates and, often, the presence of vitreous cells [8]. Pathophysiologically, this appearance results from a prelaminar vasculitis, with the retina participating as an innocent bystander. Although often described as a maculopathy, the changes in the optic nerve and not the macula are the primary cause of reduced vision in this condition [9,10]. Optic disk swelling, is the earliest sign of this disease, the macular star may be present at the onset of visual loss or may be noted only 1–2 weeks after development of the disk oedema. The macular star may even be noted only after the disk swelling is beginning to resolve. Thus, patients with acute disk swelling with a normal macula should be reexamined within 2 weeks to determine the presence of a macular star because the development of a macular star is of prognostic importance, especially in multiple sclerosis where the presence of a macular star militates strongly against subsequent development of this disease [11]. The physical exam should include, in addition to a basic ophthalmic exam, formal visual fields and imaging studies such as fundus photographs and OCT to document previously described posterior pole findings associated with neuroretinitis, as well as to follow the evolution of the posterior pole appearance [12]. The more common treatable causes must be excluded wich are cat scratch disease (CSD) and vascular disease [2]. Other infectious causes include [12]: Syphilis, Tuberculosis, Salmonella, Varicella, Herpes simplex and zoster, Influenza, Hepatitis, Epstein-Barr virus.

Inflammatory causes include [10]: Sarcoidosis, Systemic lupus erythematosus, Behcet disease, Polyarteritis nodosa, Takasayu's arteritis, Vogt-Koyanagi-Harada.

Fifty percent of cases have no identifiable cause and are labeled idiopathic neuroretinitis [13].

However, unlike LISN, some of these conditions are potentially vision- and even life-threatening and require careful consideration before a diagnosis of LISN is made [2] Blood pressure measurement is essential to exclude malignant hypertension. Blood glucose and erythrocyte sedimentation rate are necessary to help exclude diabetic retinopathy and arteritic optic neuropathy. A complete blood examination, including differential and morphology, may support the presence of infection and help exclude serious haematological problems that may potentiate venous occlusion.

When treating neuroretinitis, one should take into consideration the type of neuroretinitis with which the patient presents to determine which therapy, if any, will result in the best visual outcome for the patient [12]. Infectious causes should be aggressively sought because appropriate antibiotic treatment is warranted [11]. There is no consensus regarding optimal treatment [6]. Usually, no treatment is needed. Corticosteroids have been used in some cases, and the effect was unclear [11]. Patients with idiopathic recurrent neuroretinitis may be treated during acute episodes with corticosteroids and have been shown to benefit from long-term prophylactic immunosuppression, which reportedly reduces the number of disease recurrences [7].

The prognosis of Leber's idiopathic stellate neuroretinitis is good in most cases. Patients with recurrent disease may not experience pronounced improvement in optic nerve function [11].

## 4 Conclusion

In conclusion, in any patient presenting with an optic disc oedema associated with stellate maculopathy, it is important to rule out all potentially treatable differential diagnoses before retaining the diagnosis of Leber's idiopathic stellate retinopathy. The visual and sometimes even vital prognosis is at stake. Complementary examinations must be oriented according to the data of the clinical examination.

## Provenance and peer review

Not commissioned, externally peer-reviewed.

## Funding

This research did not receive any specific grant(s) from funding agencies in the public, commercial, or not-for-profit sectors.

## Ethical approval

I certify that this kind of manuscript does not require ethical approval by the Ethical Committee of our institution.

## Consent

Written informed consent was obtained from the patient for publication of this case report and accompanying images. A copy of the written consent is available for review by the Editor-in-Chief of this journal on request.

## Registration of research studies

This is a case report that does not require a research registry.

## Annals of medicine and surgery

The following information is required for submission. Please note that failure to respond to these questions/statements will mean your submission will be returned. If you have nothing to declare in any of these categories, then this should be stated.

## Please state any conflicts of interest

The authors declare no conflict of interest.

## Please state any sources of funding for your research

This study did not receive any sources of funding.

## Ethical approval

This type of study does not require any ethical approval by our institution.

## Consent

Patient provided written, retrospective consent for publication following detailed explanation of the purpose of manuscript and understanding that no identifiable information was going to be released.

## Registration of research studies

Elsevier does not support or endorse any registry.

1. Name of the registry:
2. Unique identifying number or registration ID:
3. Hyperlink to your specific registration (must be publicly accessible and will be checked):

## Guarantor

O.Nabih.

## Declaration of competing interest

The authors declare that they have no competing interests.
